# mRNA as a Therapeutics: Understanding mRNA Vaccines

**DOI:** 10.34172/apb.2022.028

**Published:** 2021-05-16

**Authors:** Ferdi Oğuz, Harika Atmaca

**Affiliations:** ^1^Section of Molecular Biology, Department of Biology, Institute of Natural and Applied Sciences, Manisa Celal Bayar University, Muradiye, Manisa, Turkey.; ^2^Section of Molecular Biology, Department of Biology, Faculty of Science and Letters, Manisa Celal Bayar University, Muradiye, Manisa, Turkey.

**Keywords:** mRNA vaccine, Antigen-presenting cell, Dendritic cells, Vaccine delivery systems, Cancer therapy

## Abstract

Vaccination is one of the important approaches in the prevention and control of diseases. Although the capacity to present antigens other than the disease-specific antigen in the traditional vaccine composition provides a potential benefit by increasing its protective efficacy, many components that are not needed for the related disease are also transferred. These components can reduce vaccine activity by lowering immunity against protective antigens. The reasons such as the low effectiveness of traditional vaccines and the high cost of production and time-consuming reasons show that it is necessary to develop a new vaccine method for our world, which is struggling with epidemics almost every year. Among nucleic acids, mRNA has many advantages, such as genomic integration, induction of anti-DNA autoantibodies, and immune tolerance induced by long-term antigen expression. mRNA vaccines have become a therapeutic target for reasons such as efficacy, safety, fast and non-expensive production. The fact that mRNA triggers both humoral and cellular immunity and goes only to the cytoplasm, not to the nucleus, makes it highly efficient. The mRNA must cross the lipid bilayer barrier and entry to the cytoplasm where it is translated into protein. There are two main ways of mRNA vaccine delivery for this: ex vivo loading of mRNA into dendritic cells (DCs) and direct injection of mRNA with or without a carrier. Studies continue to understand which delivery system is therapeutically more efficient. Preclinical and clinical trials showed that mRNA vaccines trigger a long-lasting and safe immune response.

## Introduction

Vaccines protect humanity against various diseases for many years. With the widespread use of vaccines, the spread of some diseases has decreased in the world.^1^ Conventional vaccines have been used successfully against infectious diseases for many years.^2^ Live attenuated vaccines are obtained by attenuating the pathogenic live microorganisms under laboratory conditions and are a vaccination strategy that mimics the natural infection. BCG vaccine against tuberculosis and measles, mumps, and rubella combination vaccines are examples of live attenuated vaccines. Inactivated vaccines are generally produced in the egg or cell culture and are partially safer than live attenuated vaccines, as they are formed by inactivating the purified disease agent by chemical, heat treatment, or radiation. Hepatitis A, influenza, polio, and rabies vaccines are examples of inactivated vaccines. Toxoid vaccines are obtained by inactivating bacterial toxins by chemical or heat treatment. Commonly used toxoid vaccines are those produced from Corynebacterium diphtheria for diphtheria and *Clostridium tetani* for tetanus. Conventional vaccines have many disadvantages, such as the risk of re-virulence of live attenuated vaccines and the risk of infection in those with immunodeficiency, inactivated vaccines being less effective than attenuated vaccines, and commercial vaccines based on toxoids that require complex components in the culture medium.^3-7^ For this reason, the development of alternative vaccines is necessary for both cancer and infectious diseases.

As a result of the advancement of science and technology, mRNA has become the therapeutic target in the fields of vaccine development. mRNA vaccines are promising for many diseases due to their efficacy, safety, fast and non-expensive production.^8^ mRNA contains only the components directly required for the expression of the encoded protein. The fact that mRNA is not replicative and degrades in the cellular process makes it safe. The encoding and expression of proteins by mRNA contribute to the development of therapeutic vaccines for the treatment of various diseases.^9,10^ T cell fatigue is prevented as antigen expression is not permanent after vaccination. Since the RNAs are functional in the cytoplasm, they do not enter the nucleus, which means that the activity of the mRNA will be higher.^11^ mRNA has its adjuvant properties, making them superior to polypeptide and protein-based vaccines.^12,13^ Due to the chemical structure of the mRNA sequences, the possibility of integrating mRNA into the host DNA genome to trigger an immune rejection reaction is low.^14^

Severe acute respiratory syndrome (SARS), Middle East respiratory syndrome (MERS), Ebola, and finally COVID-19 outbreaks have shown how quickly infectious diseases spread. In recent years, there have been significant advances in mRNA vaccine investigation.^15,16^ The design of mRNA sequences and the development of safe mRNA vaccine delivery materials with high efficacy have contributed to significant progress in vaccine investigation.^17^

In this review, mRNA vaccine investigations, application areas, mRNA vaccine delivery materials, clinical trial applications of the vaccine, advantages, and disadvantages of mRNA vaccine compared to other vaccines are discussed.

## What are nucleic acid vaccines?

Nucleic acid vaccines (NAVs) contain antigens encoded by DNA or RNA. Transfection of the DNA vaccine vector, located in the recombinant bacterial plasmid backbone containing the eukaryotic cell promoter and genes encoding the protective antigen, into eukaryotic cells is the first target in the DNA vaccine. Presentation of the encoded vaccine antigens with antigen-presenting cells (APCs) through pathways after transcription and translation steps triggers the cellular and humoral response. DNA vaccines have a limitation that prevents human clinical trials due to their low transfection efficiency, the need for booster doses, and low immunogenicity. The relatively low immune response can be increased with new adjuvant systems created using nanotechnology. Since exogenous DNA has a risk of integration into the host genome, it can lead to serious mutagenesis and new diseases. DNA vaccine studies continue today to eliminate all these risks.^18,19^

RNA vaccines include mRNA synthesized by in vitro transcription using bacteriophage RNA polymerase and template DNA encoding associated antigens. After being applied to the host cell and adopted by the cells, mRNA transcripts are translated into the cytoplasm. The resulting antigens interact with APCs to trigger an immune response.^20^ NAVs are very important as they enable multiple antigens to be administered with a single vaccine and trigger both humoral and cellular immunity.^20^ It has been found that DNA vaccines produce fewer immune responses than cellular vaccines, RNA vaccines, peptide-based vaccines, and many other vaccines. The reasons for this can be stated as inefficient transport of DNA, low DNA expression, DNA passing through both the cell and nuclear membrane, and inefficient transport of DNA. RNA does not enter into the nucleus as DNA does, it entries into the cytoplasm where translation occurs and does not integrate into the genome.^20^ For these reasons, RNA vaccines have become more attractive than DNA vaccines.^20,21^

## mRNA

### mRNA structure and synthesis

The mRNA consists of 5’ cap, 5’-UTR (untranslated region), stop signal encoding sequence, 3’-UTR, and poly-A tail. mRNA provides the template in the cytoplasm of the cell for translation into the protein encoded by the ribosome and tRNA. As a result, multiple copies of the protein are obtained from one mRNA template.^22^

Functional synthetic mRNA is achieved with in vitro transcription of the cDNA template utilizing bacteriophage RNA polymerase. Therefore, the first step in mRNA production is the preparation of pcDNA (plasmid DNA). pcDNA includes structures of bacterial genomic DNA. Therefore, the pure pcDNA required for the vaccine must be prepared in a reproducible manner. When linearized pcDNA is transcribed utilizing bacteriophage RNA polymerase, the heterogeneity of bacterial DNA residues and pcDNA is not a problem because the DNA is removed in later stages. The pcDNA template for in vitro transcription contains bacteriophage promoter, open reading frame (ORF), and restriction site. The linearized pcDNA template is transcribed in a mixture including recombinant RNA polymerase and nucleoside triphosphate. Regular cap analogs can be incorporated into the reaction to obtain capped mRNA by transcription. The cap can also be joined enzymatically after transcription. After transcription, the pcDNA template and bacterial DNA are digested by the DNase enzyme.^9^

### Purification of mRNA

During the translation phase, mRNA purity is important for determining protein yield and stability.^23^ Contamination with dsRNAs (double-stranded RNA) due to abnormal RNA polymerase activity hinders the translation and degradation of cellular mRNA and ribosomal RNA. The removal of dsRNAs raises translation significantly.^24^ Lithium chloride (LiCl) was used for this purpose in the past, but it did not remove dsRNAs. Purification by fast protein liquid chromatography (FPLC) or high-performance liquid chromatography (HPLC) can be used to remove any product and produce mRNA on a large scale.^25^

Also, codon optimization, which is an important method to refrain from unused or underused codons, increases protein production and mRNA stability.^8^ Sequence and secondary structures created by mRNAs can be identified by innate immune receptors, which can hinder protein translation. Methods such as sequence optimization and modified nucleosides can be used to raise the efficiency of vaccines.^26,27^

### Design of mRNA sequence

Poly-A tail, UTR, and 5’ 7-methylguanosine triphosphate (m^7^G) cap are essential for mRNA stability and translation.^28^ Because mRNA stability and efficiency of translation affect the amount of antigen, which determines the degree of an immune response.^20^

Capping of in vitro synthesis (IVT) mRNA transcripts is performed using cap analogs. It has been found that mostly cap analogs interact with the proximal methylated G to the RNA by reverse orientation. Therefore, approximately a third of the mRNA molecules cannot be methylated at their cap. mRNA deficient methylation of the cap base is not translated. To solve this problem, anti-reverse cap analogs (ARCA) have been designed to interact in the correct orientation. In ARCA, 3’-O-methylation of the base-methylated guanosine only lets joining a nucleotide to the non-methylated guanosine.^1,9^ ARCA-capped mRNA transcripts have both higher translational efficiency and long-term preserved high protein expression in cells transfected with ARCA-capped IVT mRNA transcripts. Protein expression from mRNA that is capped and transcribed in vitro can be increased by 2’-O-methylation of the first transcribed nucleotide. This result is similar to protein expression from mRNA transcriptionally capped with ARCA.^20,29^

A poly-A tail can be added to the IVT mRNA by encoding the poly-A tail in the DNA from which the mRNA is transcribed or using recombinant poly-A polymerase. Studies show that elongation of the poly-A tail increases the level and efficiency of protein expression.^1,30^ Poly-A tail elongation also increases the efficiency of polysome formation.^31^ The translation of the in vitro transcribed mRNA transfected into cultured cells extended the poly-A tail (from 54 to 94 residues). Peak protein level doubled when the poly-A tail was elongated from 64 to 150 residues. Further lengthening of the poly-A tail by enzymatic polyadenylation resulted in a further increase in protein expression.^9^

IVT mRNA is optimized by the inclusion of the 5’- and 3’- UTR that have been found to increase RNA stability and translational efficiency. The best-known examples of such UTRs, beta-globin 5’-and 3’-UTR increase translational efficiency, while alpha-globin 3’-UTR stabilizes mRNA.^20^ Xenopus beta-globin 5’- and 3’- UTRs were found to provide better translational efficiency on heterologous mRNA in the mouse NIH 3T3 fibroblast cell line.^9^ A combination of alpha-globin, known to stabilize mRNA, and beta-globin, known to increase translational efficiency, has been utilized to create a library of tumor-based cRNA to develop vaccines against metastatic melanomas.^32^ UTRs of non-globin genes have been incorporated into mRNAs transcribed in vitro to research the therapeutic value of mRNAs. It has been found that the 5’-UTR of the tobacco etch virus increases the in vitro transcribed mRNA translation efficiency in mammals.^9^ Also, a construct of the 5’-UTR of human heat shock protein 70 increases the translation efficiency of mRNA in mammals, so this is thought to be important for vaccine investigation.^33^ Incorporation of the internal ribosomal entry site into in vitro transcribed mRNA can enable the expression of therapeutic proteins. Vaccination with dendritic cells (DCs) transfected with non-capped mRNA containing IRS prevented metastasis in mice.^34,35^

IVT mRNA can be generated by including chemically modified nucleosides. Adding nucleotides to mammalian RNA during post-translational RNA processing in eukaryotes is being studied as a method to make IVT mRNA less immunogenic. IVT mRNA including modified nucleosides has increased translation and stability.^20^

### Basic features of mRNA vaccines

RNA vaccines are divided into conventional mRNA vaccines and self-amplifying mRNA vaccines (SAM). Traditional mRNA vaccines encode only the relevant antigen, while SAM vaccines encode a designed RNA virus genome.^36,37^ RNAs called replicon are used to provide high levels of expression of the antigen gene in host cells. Since replicons do not have viral protein genes, they do not produce infectious virions and spread to other surrounding cells. Alphavirus-based replicons contain ORF and non-translated regions. The ORF at the 5’ end encodes polyproteins translated from genomic RNA processed into non-structural proteins (nsPs) with various functions.^38^ Other ORF express the antigen that replaces the viral protein by being translated from subgenomic RNA. Replicon particles can be packaged with cell cultures or delivered with delivery vehicles.^36,39^

**Figure 1:**
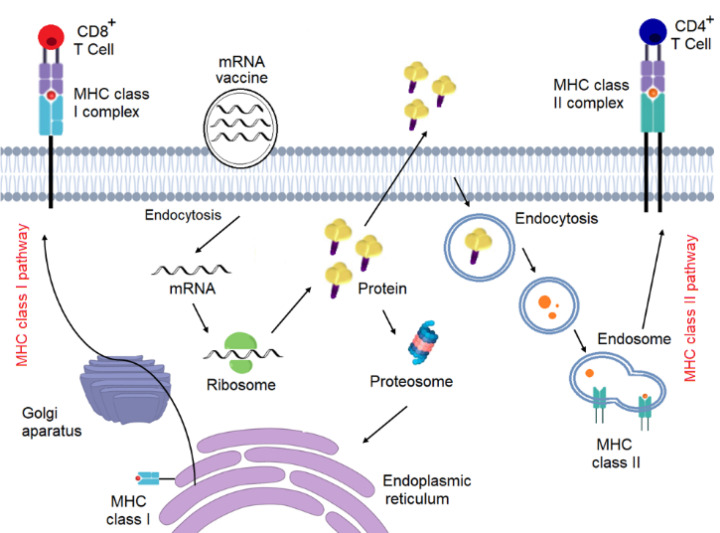
Mechanism of action of mRNA vaccines.

mRNA vaccines can trigger both humoral and cellular immunity (Figure 1). During a viral infection, T cells and B cells induce both cell-mediated immunity and antibody-mediated immunity, respectively. In cell-mediated immunity, cytotoxic T cells kill infected cells, while antibodies neutralize the virus itself in antibody-mediated immunity. While mRNA vaccines harmlessly mimic the virus’s ability to trigger the body’s immune responses to infection and elicit both types of immunity.

## mRNA vaccine delivery

Effective in vivo mRNA delivery is a crucial step for the therapeutic suitability of the vaccine. Exogenous mRNA must cross the lipid bilayer barrier to reach the cytoplasm where it is translated into a functional protein. There are two main routes of delivery of mRNA vaccines: ex vivo loading of mRNA into DCs and direct injection of mRNA with or without a carrier.^40^ Ex vivo DC loading is advantageous in terms of cellular target, transfection efficiency, but it is an expensive and difficult method. Although direct mRNA injection is fast and inexpensive, the lack of efficient cell type-specific delivery is its disadvantage, however, there have been many studies recently to overcome this problem.^41^

DCs that initiate the immune response by internalizing antigens and presenting them to CD8 and CD4 cells on the major histocompatibility complexes are the strongest APC cells. For these reasons, it appears to be very attractive for mRNA vaccines. Electroporation enables the mRNA to pass through the membrane pores and directly reach the cytoplasm, which increases transfection efficiency.^42,43^ This delivery method is very attractive as it provides high transfection efficiency without the need for a carrier. DCs loaded with mRNA are re-inoculated into the recipient to trigger the immune response. Ex vivo loaded DC vaccines can be utilized as a therapeutic target in cancer, as they induce cell-mediated immune response.^40^ Vaccine methods using DCs transfected with tumor-associated antigen (TAA) mRNA can inhibit the growth of patient-specific tumor cells.^20^

This method of vaccination reduces the risk of autoimmune triggering in patients by the addition of normally expressed endogenous proteins. The negative aspects of this method are that the vaccine development cost is high and that not all TAAs elicit an antitumor response. Many investigations have used TAA mRNA complex DCs to trigger an antitumor response.^20,40,44^ In one study, DCs were implemented to prostate cancer patients after being transfected with prostate-specific antigen (PSA) TAA, and PSA-specific T cell response was induced in six of seven patients. In another study, CEA (carcinoembryonic antigen) mRNA complex DCCs were used to vaccinate patients with tumors expressing CEA, and the antitumor response was detected in only 6 of 24 patients.^20,45^

For high efficiency of mRNA uptake, it is possible to penetrate the cell membrane by physical methods. A study has shown that mRNA complexed with gold fragments is expressed in tissues with a gene gun. Although the gene gun has been reported to be an effective method of RNA vaccination in mice, there is no data on its efficacy in humans. One study reported that electroporation improves the therapeutic efficiency of SAM vaccines, not non-replicated mRNA vaccines. Physical methods may be insufficient at the point of cell death and reaching tissues. This problem can be avoided by using lipid or polymer-based nanoparticles.^1,46^

Whereas the cationic peptide protects the protamine mRNA from RNases, insufficient protein expression has been detected in a protamine -mRNA complex cancer vaccine model.^1,9,10^ This problem has been resolved with the evolution of the RNActive vaccine platform.^47^ Although cell-penetrating peptides (CPPs) are not used much in mRNA vaccines, there have been some important advances in recent years. CPPs containing Arg-Ala-Leu-Ala motifs have been improved to concentrate the mRNA into fragments that degrade and penetrate the cell membrane. It was observed that mice obtained strong T cell responses after being vaccinated with CPP complex RNA.^48^

Highly effective mRNA transfection reactivates based on cationic lipids and polymers exist, although they show therapeutic efficiency in cancer cells, they show limited efficacy or high toxicity.^24^ Cationic lipids and polymers, including dendrimers, are widely used in mRNA vaccine application in recent years. Lipid nanoparticles (LNPs) are composed of ionized cationic lipid, lipid-linked polyethylene glycol, cholesterol, and phospholipid components.^1^ mRNA LNP complex targets the liver wherefore the binding of apolipoprotein E and uptake by hepatocytes through the receptor. Prolonged and highly effective protein expression has been detected at the injection region.^49-51^

The efficiency and duration of in vivo protein manufacture of mRNA LNP complex vaccines can be controlled by varying the route of implementation. It has been found that intramuscular and intradermal administration of the mRNA LNP complex caused more permanent protein expression.^50^ These properties of the mRNA LNP complex can be an important step in triggering the immune response. Some scientists have used lipids and polymers to deliver mRNA vaccines against HIV-1 to elicit HIV-specific CD4 and CD8 T cell responses and to detect antigen-specific immune response.^52^ Combining LNP with nucleoside modification increases the therapeutic activity of mRNA vaccines. It has been found that the complex of influenza virus HA from H10N8 and H7N9 with LNP triggers a strong immune response in mice, ferrets, and monkeys.^41,51^ There is currently no vaccine available to prevent mosquito-borne Zika virus disease. However, studies have indicated that two vaccination with LNP encapsulated mRNA encoding wild type or regulated prM-E (precursor membrane envelope) gene triggers antibody production in the body.^53,54^ In Ebola virus studies, maximum protein expression has been observed in guinea pigs six hours after intravenous injection of LNP mRNA-based vaccine. This is a promising step to develop a protective vaccine against the Ebola virus.^55,56^

In another study, it has determined that LNP SAM complex vaccines encoding influenza virus antigens trigger strong T-B cell immune responses and at the same time provide protection against homologous and heterologous influenza viruses.^57-59^

## mRNA vaccines in cancer therapy

Recently considered as a therapeutic target in cancer, RNActive technology is under clinical evaluation.^60^ Studies have been conducted on E.G7-OVA tumor cells to determine the antitumor efficiency of RNActive vaccines. Mice were exposed to E.G7-OVA cells one week after being inoculated twice intradermally with mRNA vaccine expressing ovalbumin. A significant slowdown in the expansion of tumor cells was observed in mice immunized with OVA-RNActive compared to control mice.^10^

Further studies have determined that antigen-specific vaccination not only triggers the production of albumin-specific IgG1 and IgG2a antibodies but also triggers both cellular and humoral immunity. Different studies using the antigen PSMA (prostate-specific membrane antigen) have shown that the cytotoxic effect can be increased and contributes to T cell formation. The raising of PSMA-RNActive inoculation biweekly from two to four or six caused a substantial rise in both the number of IFN-gamma-secreting CD8 cells and cytotoxicity.^10^

The researchers reused EG7-OVA cells to determine the effectiveness of RNActive technology. Mice were subcutaneously exposed to tumor cells and inoculated with OVA-RNActive twice a week when the tumor grew. Although vaccination slowed tumor growth, it could not destroy tumor cells completely. RT-PCR showed that ovalbumin expression decreased or even disappeared in tumor cells of mice inoculated with vaccine expressing ovalbumin. This suggests that tumors in mRNA vaccinated mice avoided immunotherapy resulting from the decrease in ovalbumin expression.^10^

CV9103, an RNActive vaccine as a therapeutic target, has been clinically evaluated in prostate cancer patients. The vaccine was tested in phase I and phase IIa studies on 44 patients with castrate-resistant prostate cancer with a high PSA and metastatic disease patients. While 259, 640, 1280 µg of total RNA was tested in the phase I trial, it was aimed to trigger antigen-specific cellular and humoral immune responses following the injection with the highest dose in phase IIa trial. In the research, side effects such as high fever, fatigue, most of which are mild to moderate, were seen and resolved with treatment. Antigen-specific cellular immunity was detected in 76% of patients who were vaccinated with the highest dose. 58% of responding patients and 45% of patients at the maximum dose level had a response to more than one antigen. Although a rise in PSA-specific antibodies was detected in 12% of the patients, no rise in prostate stem cell antigen (anti-PSCA) antibodies were detected. The efficiency of CV9103 in the clinical study was evaluated by the progression of PSA serum levels. The average duration of PSA-associated PFS (progression-free survival) is 1.8 months, and PSA response was detected in only one patient. Investigation anticipated an average survival of 31.4 months for 36 patients with metastatic castrate-resistant prostate cancer. The result is even better in patients who respond to more than one inoculation antigen. However, the relationship between immune responses to multiple antigens and a better survival period does not indicate a direct therapeutic impact of inoculation.^61^

Whether vaccines can be used with present treatment methods is a significant question. For this reason, the researchers tested whether the vaccines could be used with chemotherapy or radiotherapy. The combination of chemotherapy and vaccine was investigated using both chemotherapeutic drugs docetaxel or cisplatin and mRNA vaccine against ovalbumin in E.G7-OVA tumor cells. After subcutaneous exposure to tumors, mice were vaccinated with RNActive vaccine, then were treated with docetaxel and re-vaccinated. This considerably decelerated tumor expansion compared to treatment with docetaxel or mRNA vaccine alone. The same outcomes were determined when cisplatin was utilized instead of docetaxel.^60^ Consistent with the results of studies using viral vector vaccines,^62^ no deceleration in tumor expansion was observed when chemotherapy was administered before inoculation.^63^ The combination of radiotherapy and vaccine was also studied in E.G7-OVA tumor cells. After the mice were exposed to the tumors, the mice were irradiated for three consecutive days. In addition to radiotherapy, mice were simultaneously vaccinated with RNA vaccines several times. A significant slowdown in tumor growth was detected in mice, even in 3 out of 7 mice the tumor was completely disappeared.^64^

Triggering the antigen-specific cellular response to the tumor-related antigen with inoculation can be important in immune checkpoint blockade. The researchers administered the mRNA vaccine with the anti-CTLA-4 (anti-cytotoxic T-lymphocyte-associated protein 4) antibody to the mice to determine the results of using the checkpoint inhibitors in combination with the vaccine. After exposure to E.G7-OVA tumor cells, mice were both immunized with vaccine expressing ovalbumin and treated with anti-CTLA-4. While the use of anti-CTLA-4 single did not affect the tumor, the combination of anti-CTLA-4 and RNActive vaccine considerably decelerated tumor expansion.^63^

Triggering of neoantigen-specific T cell activity by inoculation is promising for cancer patients. Personalized treatments present specificity for patients due to tumor-limited expression of target antigens.^60^ CAR (chimeric antigen receptor) T cells are a personalized treatment method utilizing T cells reproduced from the patient. These are designed to express receptors that enable them to identify a specific antigen and attack tumor cells. CAR T cells can be designed to target almost any TAAs.^65^ The identify of tumor antigen is free of human leukocyte antigen as CARs contain antibody binding domains specific for TAA. Therefore, the application area is wide and can be an option to eliminate tumor escape mechanisms.^66^ mRNA electroporation has been utilized to design T cells with temporary CAR expression.^67^ Two individuals, one with advanced mesothelioma cancer and the other with metastatic pancreatic cancer, were included in the phase I clinical trial. Investigators have utilized mRNA electroporation to design patient-reproduced T cells with CAR that targets mesothelin overexpressed in some cancers. Designed T cells were repeatedly implanted in patients. In addition to observing antitumor activity in both patients, CAR Tmeso cells were found to remain temporarily in the blood after intravenous implementation and migrate into tumor tissue.^68^

## Clinical Trials

Outbreaks caused by influenza viruses in the world affect many people and even cause the death of some. Due to the benefit of mRNA-based influenza vaccine could bring, there are many studies in this area. The fact that mRNA vaccine production time is shorter and easier than traditional vaccines indicates that a significant portion of the population can be protected against possible outbreaks.^69^ In 2013, Hekele et al cloned the HA gene into the DNA template of the SAM vaccine in the H7N9 influenza epidemic in China and achieved mRNA vaccine production within 8 days after seeing the HA sequence.^59^

The LNP mRNA complex has been used to develop a universal influenza vaccine that triggers a strong immune response. The most important target in this approach was the immune subdominant HA stem, which is less prone to escape mutations.^70^ Utilizing the FPLC-purified HA-expressing mRNA-LNP vaccine, Pardi et al found strong antibodies to the HA (hemagglutinin**)** stem in ferrets, rabbits, and mice. They also demonstrated protection against both homologous and heterologous influenza virus in mice.^71^ The results of the first human trial study of the nucleoside-modified LNP-mRNA-based influenza vaccine encoding the H10N8 HA antigen have been published. Exactly 43 days after vaccination with the mRNA vaccine, it was found that the vaccine was immunogenic in all subjects, and antibodies formed at lower levels compared to animal models. These results are promising for the future.^51^

In addition to the effect of mRNA vaccines on viral infections, their benefits on parasitic diseases have been investigated by using malaria as a target disease. SAM vaccine triggered both humoral and cellular immune response against PMIF (plasmodium macrophage migration inhibitory factor) and anti-PMIF immunoglobulin G (IgG) and abolished the proinflammatory effect of PMIF. Vaccination triggered the Tfh (T follicular helper cell) cell and GC response and increased the differentiation of CD4-CD8 T cells. Additionally, the study showed that mice recovered from the infection were entirely avoided against even a second threat of infection.^72^

Pardi et al and Richner et al conducted independent mRNA vaccination studies against the Zika virus, both groups found significant levels of neutralizing titers and virus protection after two 10 µg i.m vaccination or one 30 µg i.d vaccination in mice.^53,73,74^ In another study, Erasmus et al tested a self-amplifying mRNA Zika vaccine with nanostructured lipid carrier and found a persistent immune response in mice even with a single dose of 0.01 µg vaccination.^75^

In 2012, Geall et al defined the HIV-1 Env gp140 (HIV-1 envelope glycoprotein 140) SAM vaccine in mice. They obtained env specific immune responses with CD8 T cell responses using different delivery systems.^37^ In recent studies, self-amplifying RNA-LNP encoding HIV-1 Env gp140 protein implemented to mice, as a result, antigen-specific IgG response was detected. High antibody titers were obtained in mice after a single vaccination. Although the results are promising, additional studies are needed to increase antibody resistance in HIV infection treatment.^76^

In a study conducted to see the effects of mRNA vaccines on cancer, it was found that nucleotide-based prostate cancer immunotherapy with RNActive-based components such as CV9104 presents over specificity as it exposes just antigen-positive tissues to therapeutic effect. It has been observed that the self-adjuvant prostate cancer vaccine triggers the native immune system and memory cells.^77^

Liu et al created nanoparticles to design an mRNA vaccine encoding the tumor antigen MUC1 (Mucin 1, Cell Surface Associated) into DCs to activate and increase T cells in triple-negative breast cancer studies. They united the monoclonal antibody anti-CTLA-42 with the mRNA vaccine to increase the anti-tumor response. In vivo studies have shown that the NP-mRNA (nucleoprotein-mRNA) vaccine targeting mannose receptors on DCs expresses tumor antigen in DCs of the lymph nodes and can considerably increase the potent cytotoxic T lymphocyte response and antitumor immune response against triple-negative breast cancer 4T1 cells.^78^

## Conclusion

Studies on mRNA vaccines in recent years provide important information on the applicability of the vaccine. The availability of various mRNA delivery systems, rapid and easy production of the vaccine increases the interest in this field day by day. The data obtained from mRNA clinical vaccine studies for diseases such as cancer, viral infections, and bacterial infections are very promising for the future. Research is ongoing to compare nucleoside modified mRNAs against unmodified mRNAs, compare self-amplifying mRNAs against conventional mRNAs, and search for the best delivery method.

## Ethical Issues

Not applicable.

## Conflict of Interest

The authors declare that they have no competing interests.
